# Therapeutic Approaches for Metastases from Colorectal Cancer and Pancreatic Ductal Carcinoma

**DOI:** 10.3390/pharmaceutics13010103

**Published:** 2021-01-14

**Authors:** Adriana G. Quiroz-Reyes, Jose F. Islas, Paulina Delgado-Gonzalez, Hector Franco-Villarreal, Elsa N. Garza-Treviño

**Affiliations:** 1 Biochemistry and Molecular Medicine Department, School of Medicine, Universidad Autonoma de Nuevo Leon, Monterrey 64460, Mexico; he.franco@althian.com (A.G.Q.-R.); jose.islasc@uanl.mx (J.F.I.); paulina.delgadogn@uanl.edu.mx (P.D.-G.); 2 Althian Clinical Research, Monterrey 64000, Mexico; dr.hectorfranco@gmail.com

**Keywords:** lung metastasis, liver metastasis, cancer stem cells, cancer treatments and progression biomarkers

## Abstract

Metastasis is the process of dissemination of a tumor, whereby cells from the primary site dislodge and find their way to other tissues where secondary tumors establish. Metastasis is the primary cause of death related to cancer. This process warrants changes in original tumoral cells and their microenvironment to establish a metastatic niche. Traditionally, cancer therapy has focused on metastasis prevention by systematic treatments or direct surgical re-sectioning. However, metastasis can still occur. More recently, new therapies direct their attention to targeting cancer stem cells. As they propose, these cells could be the orchestrators of the metastatic niche. In this review, we describe conventional and novel developments in cancer therapeutics for liver and lung metastasis. We further discuss the resistance mechanisms of targeted therapy, the advantages, and disadvantages of diverse treatment approaches, and future novel strategies to enhance cancer prognosis.

## 1. Introduction

Metastasis is an inefficient process in which cells from the primary tumor spread by releasing circulating tumor cells (CTCs) into the vasculature towards a distant organ to colonize it, establishing metastasis. A process where only 0.01% of the cells that enter the circulation can successfully reestablish a new metastasis, and while this seems highly inefficient, it is more often than not a fatal step in cancer progression [1,2]. Cancer stem cells (CSC) are the subpopulation of cells responsible for promoting angiogenesis, local invasion, distant metastasis, and resistance to apoptosis. Moreover, epithelial tumor cells (mature population) gain invasiveness and migratory abilities through the process of epithelial–mesenchymal transition (EMT), which is where a complex network of interconnected factors and pathways meet, such as transforming growth factor-β (TGFβ), epidermal growth factor (EGF), insulin-like growth factor (IGF), WNT, Hedgehog, and Notch pathways, all of which regulate and promote CSC growth [3,4,5]. The spread of CSC from the primary tumor to a secondary site is a process highly dependent on signaling cues, such as hypoxia, acidic pH, and/or glucose deprivation [6]. However, only one out of 500 CSC will survive in the circulation, even though mechanisms to protect CSC from elimination by the immune system exist as the secretion of IL-4 and CD200, which represents an important role in immune escape [7]. CSC in the bloodstream or otherwise in the lymphatic system can cluster together with stromal cells (fibroblasts, endothelial, tumor-infiltrated myeloid cells, or pericytes) for improved metastasis potential. Endothelial cells (ECs) in healthy established vessels remain quiescent for years. Under certain conditions, such as hipoxia or inflammation, as occurs in pathologies such as cancer and wounds, they can rapidly switch to an angiogenic state and start to form new blood vessels by interaction with pericytes [8,9,10]. Once an angiogenic switch is turned on, factors (VEGF, PDGF, TNF-α, and IL-8) inside the tumor promote growth and metastasis [11,12]. A majority of ECs in the tumor vasculature are tumor-derived ECs (TECs) retain remain distinct from cancer cells, by are not immortal and their ontological endothelial identity permit participate in making up the lining of neoangiogenic vasculatures in the TME and accelerating tumor progression [13,14]. Circulating tumor endothelial cells (CTECs) with a mature phenotype, derived from vessel wall turnover, play an important role in tumor initiation, progression, metastasis, and neovascularization [15]. Moreover, it has been found clusters of endothelial cells expressing endothelial markers as vimentin and other lineage markers, such as FN1, SERPINE1, and FOXC1, that improve the survival of cancer stem cells and promote their dissemination [15]. Increased CTECs in cancer patients have a worse prognosis, indicated as potential biomarkers of angiogenesis and metastasis, and can express PDL-1 that permit the evaluation of immunotherapy efficacy [16].

Once CSC attaches and develops with success, a pre-metastatic niche will form. Pre-metastatic niches additionally require exosomes or exosomal-like extracellular vesicles (ECV) from entrained bone marrow-derived cells and macrophages to start changes in the cells. Ideally building an effective microenvironment that serves as a fertile niche for tumor cell growth [8,9,10]. Researchers estimate that between 20% and 54% of malignant tumors develop metastasis. Primarily at lymph nodes, with the liver and lungs as the second most common sites of metastasis [17]. Colorectal cancer, the third most frequent neoplasm, can progress to the liver (40–50%) and lung (10–20%) depending on the primary tumor’s stage. Clinical data show that median survival is just 5–20 months without treatment [18]. Meanwhile, pancreatic ductal adenocarcinoma (PDAC) is one of the most aggressive cancers with high metastatic potential. At the time of PDAC diagnosis, approximately 50% of patients present metastatic disease with 19–39% affected in the lungs and >50% in the liver. The median survival time is around 6–11 months in patients with metastatic pancreatic cancer [19]. Interestingly, several studies have reported that isolated lung metastases are associated with better survival outcomes than patients with other solitary metastatic organs [20,21,22]. In what follows, we will discuss differences between the cascade of events in primary tumors which promote the establishment of pre-niche and niche metastasis.

## 2. Why Liver Metastasis?

The physiological functions of the liver are related to the metabolism of nutrients absorbed by the gastrointestinal tract and the detoxification of dangerous molecules present in the circulation. These functions are responsible for a delicate balance between immune tolerance and immune response. Their correct functioning requires normal cytoarchitecture of the hepatic lobules. This cytoarchitecture includes polarized hepatocytes and an intricate network of discontinuous capillaries (sinusoids) and bile ducts. Important cell types in the lobule are Kupffer cells (KCs), a type of specialized macrophages that take up and destroy foreign material, and hepatic stellate cells (HSCs), which are involved in the response to liver damage. HSCs are found in the space of Disse, which separates hepatocytes from sinusoids [23]. Due to the total blood volume that circulates through the liver, about 30% of blood volume per minute, it is the preferred organ for metastasis development [24]. The portal vein is a vessel that carries blood from the gastrointestinal tract, gallbladder, pancreas, and spleen to the liver. The right portal vein is the continuation of the main portal vein, while it separates the left portal vein from the main portal vein at an acute angle [25]. This explains why the segment of the right lobe liver is the main target of metastases. Also, the blood circulation in the colon and proximal rectum drain through the hepatic portal system, while the blood of the distal rectum goes to the lung. This vascular organization correlates with the fact that colorectal cancer prefers liver metastasis, with the lung as the second favored metastatic site [26]. However, to allow hepatic metastasis development, the liver must present damage. There are several hypotheses regarding the pro-metastatic state consequent to cirrhosis, steatosis, and nonalcoholic fatty liver disease. Some authors state that altered hepatic cytoarchitecture creates an unfavorable environment [27]. Others report that these alterations slow the passage of neoplastic cells through the microcirculation and enhance cellular-vascular contact and stress micro thrombotic phenomena through the expression of adhesion molecules, facilitating metastasis [28]. Chronic inflammation that results from receiving nutrients, toxins, and microorganisms via the portal vein from the gut can activate HSCs or produce oxidative stress that leads to liver injury creating a microenvironment favorable for the increased growth of metastases [27]. Also, organotropism is regulated by multiple factors, organ-specific niches, and the interaction between tumor cells and the host microenvironment [29].

### Factors Crucial for the Formation of Liver Metastasis

**Figure 1:**
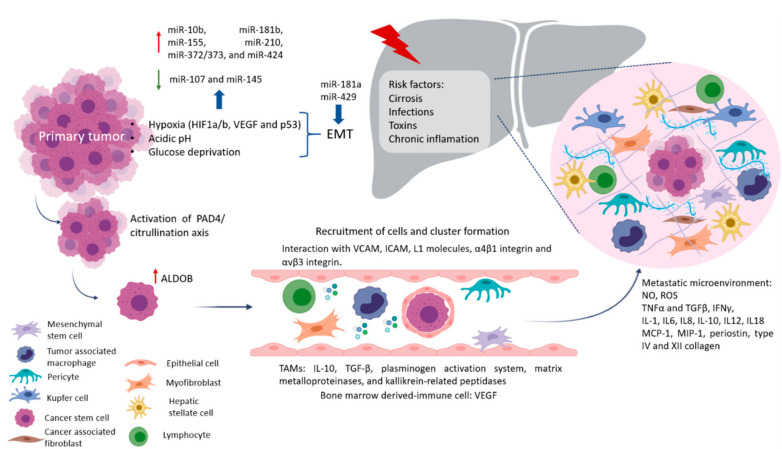
Liver metastases process. Development of liver metastases involves changes in tumor cell metabolism, as well as EMT and is induced by inflammatory cytokines, miRNAs, hypoxia, and pH. These factors allow for dissemination of CSC. Growth factors promote survival of cells in the blood and the lymphatic circulation, these cells form clusters (fibroblasts, endothelial, tumor-infiltrated myeloid cells, or pericytes) in order to arrive to hepatic tissue. Also, liver metastasis niche is improved by damage inducers, that activate HSCs, KCs, CAFs, myofibroblast, TAMs or produce oxidative stress and cytokines production promoting CSC establishment and growth.

Pre-niche is formed by the preconditions in specific organs in terms of nutrients, extracellular matrix, and immune cells necessary to generate fertile soil before the arrival of CTC, which increases the success of metastasis establishment, as shown in Figure 1 [30].

After that, primary tumors can induce stromal cell remodeling of the extracellular matrix which takes care of cell organization. To increase the survival of CTC from the hostile microenvironment, they usually form CTC clusters. Interactions between cancer cells and endothelial cells with an endothelial layer, and the underlying basement membrane VCAM, ICAM, or L1 molecules, expressed by the vascular endothelium, in interaction with α4β1 integrin and αvβ3 integrin, play a central role in leukocyte recruitment to develop this process [28,31]. Other tumor-recruited cells such as cancer-associated fibroblasts (CAFs), stromal myofibroblast, endothelial cells, pericytes, diverse immune cells, mesenchymal stem cells (MSCs), and tumor-associated macrophages (TAMs) contribute to tumor growth and serve as a prerequisite for tumor cell invasion and metastasis [8,30]. For example, during the early stages of tumor development, cytotoxic immune cells such as natural killer (NK) and CD8+ T cells recognize and eliminate the more immunogenic cancer cells. High levels of tumor-infiltrated T cells indicate a good prognosis in many solid tumors, while high levels of macrophage infiltration correlate with a worse prognosis. TAMs promote tumor progression in different ways, such as secreting cytokines like IL-10 and TGF-β, which induce immunosuppression and impair poor response by cytotoxic lymphocytes T and dendritic cell maturation [32]. Also, TAMs release a plethora of extracellular matrix (ECM) remodeling factors (plasminogen activation system, matrix metalloproteinases, and kallikrein-related peptidases), affecting the composition, structure, and elasticity of the ECM and the availability of growth factors, creating conduits for the migration of tumor cells [32]. Metastasis-associated macrophages (MAMs), in murine CRC models, have been shown to increase the extravasation of tumor cells and help in their survival by secreting growth factors and concomitantly inhibiting cytotoxic T cells [33]. Some key mediators in regulating immune responses are myeloid-derived suppressor cells (MDSC) which express CD11b+ and CD33+. CD11b is a subunit of the integrin adhesion molecule, which expresses in bone marrow-derived immune cells. CD11b was reported to promote myeloid cell migration to the tumor microenvironment, which secreted cytokines related to tumor growth and angiogenesis. Researchers relate the expansion of MDSCs to tumorigenesis in CRC, and the density of CD33+ MDSCs in the microenvironment was a negative prognostic factor in CRC patients. Recently reported higher expression (lymphocytes CD3+) in primary tumor than in hepatic metastases, while CD33 had higher expression in hepatic metastases than in primary tumor. In turn, CD33+ showed that more immunosuppression of cells in the liver may contribute to the poor response to immunotherapy [34,35,36]. Although to date, some cell types have been associated with better or worse prognoses of primary tumors, unlike in metastasis where there are many other factors involved, such as the treatment they are receiving, this may change the microenvironment and therefore favor the progression of the disease. In most patients, physicians do not focus the treatment used on destroying the cells responsible for tumor chemoresistance and progression, and therefore this approach is being increasingly included in patients with already advanced stages, increasing their survival. MicroRNAs (miRNAs or miR) are short 18–22 nucleotide non-coding RNAs that control several processes by regulating gene expression. miRNAs can bind directly to mRNAs forming miRNA-mRNA complexes at the 3′UTR, where they recruit the ribonucleotide silencing complex (RiSC) and undergo either deadenylation and degradation via CAF1 and PABP, or transcriptional repression [8,37,38,39,40,41]. Colon cancer metastasis regulation has been a widely studied topic; in particular, the study of miR which acts as oncogenes have gained track in the last couple of decades. Cancer alters miRNA expression as stress and other microenvironmental factors changed through cancer’s development [42]. Factors involved in tumor progression and the development of metastasis include hypoxia, which in CRC is very common and becomes more aggressive, invasive, and resistance to chemo- and radiotherapy. It associates with the activation of transcription factors involved in the maintenance of EMT/CSC phenotypes and enrichment of CD44^high^, CD24^low^ or ALDH^high^ CSC population [43,44]. Non-coding RNAs play critical roles in the response to hypoxia in various cancers as miR-10b, miR-181b, miR-155, miR-210, miR-372/373, and miR-424 become upregulated under hypoxia [45]. A recent review by Gonzalez-Villareal in colorectal cancer metastasis emphasized EMT regulation, with members of the miR-200 family upregulated by Ascl2 as key regulators. Moreover, miR-199a downregulation and miR-210 and miR-21 upregulation are also associated with EMT enhancing HIF-1α/VEGF expression [30]. An EMT major driver is hypoxia, which involves HIF1a/b, VEGF, and p53, all of which downregulate miR-107 and miR-145, negatively correlating with p70S6K1 [46]. Increase expression of miR-210 via HIF-1α downregulates ephrin A3, which results in decreased migration, with an increase in glucose metabolism stimulating tumor growth [47,48,49]. Particularly in liver metastasis, p53 mutations, such as R175, G245, R248, R249, R273, and R282, produce gain-of-function and typically translocate with p63 (DNp63a) and p73 to promote TGF-β by RhoA or BRAF. miR-527/665 suppresses KSRP and miR-198, switching off SMAD4 and TGF-βR2 [50,51,52]. Malignant cells have glucose consumption rates about 200 times higher than normal cells; hence, their energy has more to do with the Warburg effect. According to this model, cancer cells get energy from lactate, free fatty acids, and ketones generated from the activation of anaerobic glycolytic and autophagic programs. Several authors have proposed that CSCs possess unique metabolic features when compared with the differentiated bulk of tumor cells, and with normal stem cells which allow CSC maintenance and dissemination. This might represent an effective approach to ablate the cells at the origin of the cancer. Their nutrients become linked via metabolic networks, enhancing the ability of cancer cells to survive and seed in a certain environment. Researchers have shown that liver metastasis regulation has a dependency on miR-885 overexpression by targeting CPEB2 and vWF. Significantly, these targets involve insulin regulation via insulin-like factor growth protein 5. IGFBP5 has been shown to regulate cell growth, differentiation, apoptosis, and metastasis. Yet, oxidative phosphorylation, due to hypoxic conditions, remains a preferred energy source [30,53]. Implied in this alternative energy metabolism is creatine kinase brain-type (CKB), necessary to produce phosphocreatine and maintain ATP levels, which are secreted into the microenvironment by the downregulation of miR-483 and miR-551a [54]. In liver metastasis of CRC, researchers observed an enhancement of fructose metabolism via the upregulation of enzyme aldolase B (ALDOB), hence providing extra fuel for metastatic outgrowth [55]. Thus, as the metabolic center of the entire organism, the liver appears to provide a unique milieu enabling or forcing cancer cells to assume specific metabolic activities for colonization. It involves molecular pathways for liver colonization, including nitric oxide and ROS, and the expression of adhesion molecules such as selectins and integrins. Also involved are phagocytosis, cytokines, such as TNFα and TGFβ, interferon gamma (IFNγ), interleukins (IL-1, IL6, IL8, IL-10, IL12, and IL18), growth factor monocyte chemoattract protein-1 (MCP-1), and macrophage inflammatory protein (MIP-1) released by KCs and HSCs [56]. Proinflammatory cytokines such as IL-6, preparing a permissive inflammatory pre-metastatic niche where circulating CRC cells can home and survive, colonize, and eventually form micro and macro metastasis. IL-6 then activates signal transducer and activator of transcription 3 (STAT3) signaling in hepatocytes, which produces serum amyloid A protein (SAA) that orchestrates the formation of a pro-metastatic niche in the liver. It has been reported that S1PR1–STAT3 upregulation in tumor cells induces IL-6, which activates S1PR1–STAT3 in MDSCs in the liver, leading to pre-metastatic niche formation prior to CRC cell arrival [57]. Expression of fibronectin and granulin by macrophages stimulates HSC for differentiation into myofibroblasts, whose release periostin, creates a fibrotic environment in the liver that sustains tumor growth [58]. Similarly, human hepatic sinusoidal endothelial cells in vitro express macrophage migration inhibitory factor (MIF-1), which improves EMT, migration, proliferation, and apoptotic resistance in CRC cells. Finally, angiopoietin-like 6 protein from the liver sinusoidal endothelial cells (LSEC) induces liver colonization of CRC cells and correlates with CRC progression in in vitro models [59].

Recent works reported that type XII collagen was the most significantly upregulated collagen in cancer liver metastasis (CLM) and that there was a difference with the ECM of the CRC. Collagens that are present in metastatic disease were COL10A1, COL12A1, COL 14A1, and COL15A1. Also, collagen type IV has a strong association with liver metastasis in CRC. One important miR is miR-19, which inhibits Transglutaminase-2, a critical crosslinking enzyme of the ECM, hence inducing invasiveness [60,61]. Another tumor suppressor miR is miR-155 which acts over several important regulators such as CTHRC1 and TP53, involved in tumor intravastation [62,63,64]. Moreover, miR-155, alongside miR-21, miR-1827, miR-145, and miR-34a, activate Wnt/b-catenin signaling. Additionally miR135a/b, miR-494, and miR-19a suppress APC and directly activate Wnt [65]. Wnt regulation is paramount during metastasis, as described in this review. Another study by Ji et al. confirmed that miR-181a promotes EMT by hindering WIF-. Furthermore, miR-429, was shown to impede apoptosis and induce EMT by targeting SOX2 [66]. The potential targeting of miR-429 would then lead to apoptosis. This could be critical to “kill off” metastatic cells, although research is warranted in order to have precise control as not to lead a systemic proapoptotic process. Some factors influencing liver metastasis on CRC were found, but we know that tumor cells themselves, stromal cells, and the interaction between CRC cells and their microenvironment, all contribute to hepatic invasion. Until now, we have known much about the establishment of the pre-metastatic niche but little about the metastatic niche itself. Therefore, the search for mechanisms and possible therapeutic targets that can reverse or change the prognosis of the disease is currently being continued since most patients at the time of diagnosis already arrive in very advanced stages of the disease, when the approach to treatment is more complex than just treating the primary tumor and preventing metastasis. Next, the management of patients with liver and lung metastases in two neoplasms that are on the rise without hereditary history will be reviewed.

## 3. Lung Metastasis

The lungs are one of the most complex organs in the human body. Their function is to exchange oxygen from the external environment with carbon dioxide from the cardiovascular system. Its role in the development of metastasis can result after physical trauma that induces local inflammation (smokers, asthma, obstructive pulmonary disease, or pneumonia) and tissue damage, creating a favorable environment that attracts metastatic cells from distant sites. Also, it has been reported that lung inflammation in smokers or in people with lung diseases such as asthma, chronic obstructive pulmonary disease, and infections, such as pneumonia, are risk factors for lung metastases. Some factors related to the migration of cells and vascular permeability are CCL2, S100A8, and SAA3. Upregulation of fibronectin colocalized with LOX, and higher levels of expression of VCAM-1, a receptor for VLA-4 in patient samples of metastatic tumor nodules in the lungs and bone, have been found [67]. The pro-metastatic effect played by exposure to smoking is related to the activation of the ubiquitin-chemokine receptor type 4 (CXCR4) pathway, high tissue levels of E-selectin, activation of the nuclear factor kappa-light-chain-enhancer of activated B cells (NFκB), signaling in pneumocytes, increased chemokine ligand 2 (CCL2) expression, and macrophage infiltration in the lung microenvironment. Moreover, lung alveolar cells induce chemokine secretion, which recruits neutrophils. The latter, through the synthesis of arachidonate 5-lipoxygenase (ALOX5)-dependent leukotriene, may promote survival and proliferation of leukotriene B4-expressing metastatic clones [17]. Inflamed lungs recruit neutrophils to release Cathepsin G and neutrophil elastase and destroy the protein Thrombospondin 1 (Tsp-1), which protects lung tissue from metastasis. Another alternative, but not mutually exclusive, is that micro-metastatic foci are already present at the time of physical injury [68]. Therefore, it is believed that lung metastases depend on circulatory system involvement, which flows into the pulmonary arterial system through the subclavian vein via the thoracic duct from the lymph nodes invaded by tumor cells [69]. More recently, DPC4 loss is shown to be associated with EMT, tumor progression, and the presence pulmonary metastases [70].

### Factors Crucial for the Formation of Lung Metastasis

**Figure 2:**
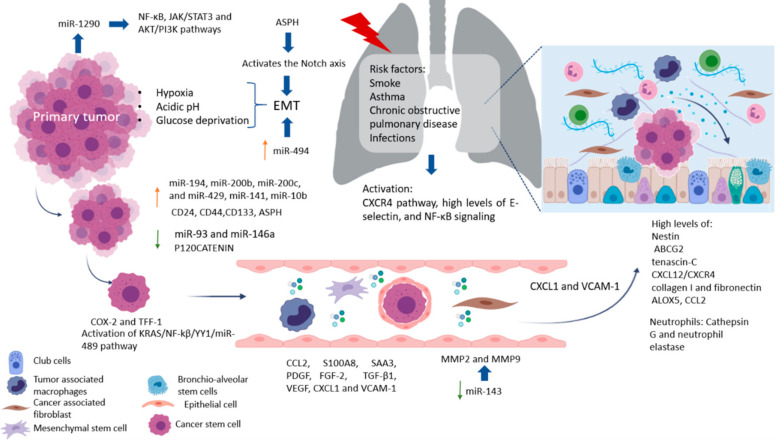
Lung metastases process. Tumor cell dissemination to lungs results after tissue damage that induces local inflammation and improves metastatic environment. club cells, alveolar cells recruit neutrophils, CAFs, TAMs, MSCs, and form a protective niche for help to CSC. In addition, metastases are promoted by CXCR4 signaling, miRNAs, growth factors, hypoxia, pH and glucose metabolism. Deposition of tenascin-C, collagen I, fibronectin and particular metalloproteases 2 and 9 after cancer cells dissemination to the lung in and important feature for CSC and tumor outgrowth.

Factors produced by cells in pre-niche can support survival and growth of disseminated tumor cells were in Figure 2. The interstitial space is the main site for lung metastasis, which is adjacent to the terminal bronchioles. Bronchioalveolar stem cells (BASC) are situated in the terminal bronchioles and present pro-surfactant apoprotein-C (SP-C), a marker for alveolar type II (AT2) cells, and CC10, a marker for club cells—both are related with the lung pre-metastatic niche. Bone marrow cells, club cells, and alveolar macrophages are also present in pre-metastatic lung [67].

Besides promoting the outgrowth of tumor cells, some microenvironmental components can suppress metastasis formation. Bone morphogenic protein (BMP) signaling is an example of an active process that can render tumor cells dormant in the lung. BMPs belong to the TGF-β family in the lungs, but not in the bone or brain. Endogenous BMP4 generated by epithelial cells and mesenchymal cells confers dormancy on metastasizing tumor cells [67]. The involvement of the tumor microenvironment contributes to invasion and chemoresistance, where the extracellular matrix, CAFs, activated pancreatic stellate cells (PSC), MSCs, and TAMs form a protective niche for CSC [21]. The origin of CSC in the pulmonary metastatic niche has been associated with the tumor microenvironment to support cell persistence and maintenance of the undifferentiated state characterized by the expression of markers such as CD24, CD44, and CD133, which are identified as resistant to treatment markers and CSC. For example, one miRNA recently studied in PDAC was miR-93, known to have implications in lung metastasis. miR-93 seems to regulate microtubule dynamics by controlling the expression of YES1, CRMP2, and MAPRE1. Dysregulation of these proteins consequently leads to growth by faults in G2/M cell cycle progression [39,71]. As cross-regulation is crucial in metastasis, recent research suggests that EGF and miR-146a have a reverse dependency in metastasis, since miR-146a levels diminish as metastasis develops. A study by Mess et al. found that highly metastatic pancreatic cells are distinct in their overexpression of miR-194, miR-200b, miR-200c, and miR-429, all of which work in concert to target the tumor suppressor gene EP300 and mutations in CREBBP [72,73]. The role of these miR-200 family members, including miR-141, is critical in EMT facilitating invasion. Likewise, miR-494 is important in EMT by targeting PTEN and TGF-β/SMAD pathways [74]. In contrast, functional studies have currently found that both miR-141 and miR-429 inhibit tumor development [75,76,77,78]. Researchers have also found that miR-10b increases invasiveness in metastasis by effecting EGF and TGF-β signaling through SMAD regulation implicating reduction of miR-323-3p and miR-193a, a decrease of both miRNAs enhances colonization by PANC-1 cells. Out of the many targets regulated by miR-10b both HOXD10 and KFL4 have implications in the control of matrix metalloproteinases -2,-9,-14 and E-Cadherin which involve extracellular matrix remodeling, helping promote angiogenesis development. Therefore, it is suitable to point out that miR-10b is a likely therapeutic target [79]. Interestingly, miR-10b is a metastatic regulator in both pancreatic and colorectal cancer, and other cancers such as lung cancer [80,81]. Therefore, a proposal would be a reactivation of miR-323-3p to control epithelial-mesenchymal transition and reduce PANC-1 cell growth [82,83,84]. In lung metastasis, nestin and ABCG2 levels are elevated [85,86] and associated with the formation of spheroids that are characteristic of CSCs. Loss of P120CATENIN has been associated with lung metastasis [85,87]. Aspartate-β-hydroxylase (ASPH) is an enzyme that can propagate an aggressive phenotype characterized by EMT, invasion, degradation or remodeling of the extracellular matrix, angiogenesis, stem and colonization to distant sites. ASPH works by activating the Notch axis. ASPH stabilizes Notch receptors, JAG ligands and the ADAM regulator improve interactions between receptors and Notch ligands, activate the Notch cascade, positively regulate the genes responsible for EMT, and recently, in PDAC models, their expression in lung metastases [88]. Pancreatic CSC has been reported to require platelet-derived growth factor (PDGF) for proliferation and induction of migration. Also, basic fibroblast growth factor (FGF-2) and TGF-β1 are required for component synthesis. Other factors are involved in the interaction of cancer cells and CSC include cyclooxygenase (COX)-2 and as the conditioned environment of cancer cells increases, COX-2 expression and CSC proliferation, and Trefoil factors (TFF)-1, whose expression stimulates the proliferation and migration of CSC [89]. The arrival of cancer cells to the lung generates a series of changes that help create mature metastatic niches. One of them is the deposition of the ECM component tenascin-C after dissemination of cancer cells to the lung which is important for metastatic outgrowth. For example, one important signaling axis in pancreatic cancer metastasis is the KRAS/NF-kb/YY1/miR-489. Recently, researchers found that KRAS activation (90% of pancreatic cancers) leads to miR-489, which exerts control over the ECM. When KRAS activates it signals YY1, suppresses miR-489, a suppressor of metastasis, leading to a signaling cascade that activates ADAM9 and MMP7 promoting migration [90]. Also involved with KRAS regulation is miR-143 which downregulates the activity of MMP2, and MMP9. Recent therapeutic research showed that restoring either miR-143 or miR-145 significantly inhibited pancreatic metastasis [73,80]. Another study by Ta et al. showed that NF-kβ, constitutively active in pancreatic cancer patients, is promoted by miR-1290, relates to the expression of IKK1 [91], and is implicated in the downregulation of miR-146a, suggesting its role as a tumor suppressor in most cancers. Meanwhile, miR-27a becomes highly upregulated and could be a target for therapeutics during metastasis. Moreover, it targets Sprouty2 which antagonizes the RAS/MAPK signaling pathway [92]. miR-27a can induce the suppression of PHLPP2, leading to stimulation of the AKT/GSK3β pathway, reducing the expression of FOXO1 and upregulating Bcl-2, which leads to minimizing cell damage [93] with less reduced damage. Apoptosis signaling is further reduced as miR-320 and miR-365 become upregulated, particularly miR-365 targets SHC-1 and blocks the pro-apoptotic regulator BAX [94]. Also, miR-1290 is responsible for downregulating the suppression of cytokine signaling 4, which leads to the activation of both JAK/STAT3 and AKT/PI3K pathways (proliferation) [95]. In addition, these patients have elevated levels of miR-31-5p, miR-101-3p, miR-34a-3p, miR-21-5p, and miR-155. Interestingly, both miR-1290 and miR-1246. because of their detectability in serum, are being studied as potential biomarkers [91,96]. In metastasis, miR-34a is found downregulated since it is a direct target of TP53 to reduce pro-inflammatory cytokines. Its restoration could be key in tumor suppression [97,98]. CXCL1/2-expressing tumor cells can recruit CD11b+ Gr1+ bone marrow cells and CXCL1/2 expression promotes lung metastasis and may contribute to metastatic niche formation after tumor cell dissemination [67]. Coculture of CSC with a subpopulation of endoglin-expressing pancreatic cancer cells (CD105) has been reported to increase the proportion of CD105-positive cancer cells and these cells present higher migration activity compared to CD105-negative cells. Additionally, the CXCL12/CXCR4 axis is implicated in the homing of cancer cells to metastatic sites which have been reported associated with melanoma and breast cancer. CXCL1 modulates the tumor microenvironment (macrophages, fibroblasts, neutrophils, and osteoclasts). Higher levels of CXCL1 are associated with tumor size, advancing stage, depth of invasion, and patient survival in liver metastasis of colorectal and hepatocellular cancer. Promotion of lung metastasis also is induced by secretion of CXCL1 and VCAM-1 expression and this regulates trans-endothelial migration of cancer cells. Elevated circulating levels of VEGF and CXCL1 are predictive of liver and lung metastasis, respectively, making them interesting as a therapeutic target for improved patient care and the prevention of many deaths from cancer [99]. Although we know a lot of factors that are involved in the establishment of the metastatic pre-niche, we still have much to discover about the niche that promotes the progression of metastasis and the best therapeutic targets or biomarkers of clinical response according to the progression of the disease. The treatment approaches and their advantages will be reviewed together with the differences observed between metastases in lung and liver and between CRC and the pancreas.

## 4. Biomarkers in Metastatic Cancer

Important biomarkers of prognostic significance reported in metastatic disease in PDAC and CRC are shown in Table 1. Prognostic factors before therapy that influence success and survival in resection of lung metastases are demographics (age, gender), primary tumor characteristics (stage, histology, origin), and lung metastases characteristics (number, size of lesions, the coexistence of liver disease, lymph nodes involved) [100].

Recently aneuploidy, leading to genomic instability is known to be the most common characteristic of malignant cells, was detected on CD31+ CTECs, together with their counterpart CD31- circulating tumor cells (CTCs), together they constitute a unique pair of cellular circulating tumor biomarkers [13,102,103]. In addition, the basic helix loop helix factor Twist1 seem to be activated by hypoxia, by this effect it can induce differentiation of tumor cells into endothelial cells and promote tumor-derived vascular formation [104]. Moreover, recent studies showed that TECs could improve tumor angiogenesis by enhancing EGFR expression, by increasing its proliferation, and losing ErbB3 expression, hence inhibiting proliferation [103]. However, recent reports propose that the co-detection of CTC and CTEC subtypes can be used to predict and evaluate therapeutic effectiveness of anti-angiogenic treatments [13]. Therefore, in this field, it is necessary to continue studying comprehensively CTECs to find novel molecular target in preventing angiogenesis and development metastasis.

Other factors that influence survival are disease-free time before metastases, and different histologic characteristics of the primary and secondary tumors [100]. This indicates that a combination of clinical features and metabolic molecules improves sensitivity and specificity parameters for the establishment of accurate biomarkers. Nowadays, when liver metastases are detected in patients with advanced cancer, surgery is still the first choice. However, most of the patients with metastasis lesions at diagnosis are not suitable for surgery, and immunotherapy may improve tumor elimination and increase survival time in cancer patients. We start by addressing the highly aggressive and notoriously lethal pancreatic cancer and its metastasis. From a clinical perspective, pancreatic cancer is a highly aggressive malignancy capable of disseminating to other tissues.

Characteristically, pancreatic cancer has the potential to metastasize quickly to various locations such as the lung and liver, yet if detected early, several treatment options are available including surgery (best scenario), chemotherapy, radiotherapy, and more recently immunotherapy [77,82,83,105]. While these treatments continue to be at the forefront, a full understanding of the underlying mechanisms of how metastasis develops and novel achievements are being sought. The available regional treatments for liver metastases from CRC include surgical resection, thermal ablation, regional hepatic intraarterial chemotherapy, chemoembolization, radioembolization, and radiation therapy (RT) including stereotactic RT, as shown in Table 2 [100,101].

## 5. Novel Strategies for Liver and Lung Metastasis Treatment

### 5.1. Immunotherapy

Immunotherapy is a therapeutic strategy that has been extended to a wide variety of cancers. In this strategy, the immune system is stimulated by different mechanisms to facilitate cancer cell elimination. It uses peptides such as cytokines, growth factors, antibodies, cells, and immune checkpoint regulators to improve immune performance [114].

### 5.2. Immune Checkpoint

Cancer cells take advantage of tolerant immune regulatory mechanisms to evade elimination by T cells. Several immune checkpoints have been identified as targets that are presented in cancer stem cells as CD28/CD80 (CD86), ICOS (CD278)/ICOSL, CD27/CD70 GITR/GITRL, or co-inhibitors, such as PD-1/PDL-1 (PD-L2), BTLA/HVEM, CTLA4/CD80 (CD86), B7H3, B7H4, B7H5/HVEM, LAG3/MHC II, TIM3/GAL9, TIGIT/Nectin-2, or IDO, as shown in Table 3. An important target in immunotherapy is programmed death receptor-1/ligand-1 (PD-1/PD-1L), which are members of the CD28 and B7 families, and participate in the induction of tolerance through the regulation of T-cell activity. PD-L1 (also known as CD274 or B7H1) and B7H3 have been identified as promoters of the CSC-like phenotype, EMT, tumor cell proliferation, metastases, and resistance to therapy. However, tumoral cells take advantage of this mechanism for immune evasion and improve survival. In some cases of CRC, this strategy has demonstrated a complete response. The U.S. Food and Drug Administration (FDA) has approved three PD-1 inhibitors (nivolumab, pembrolizumab, and cemiplimab) and three PD-L1 inhibitors (atezolizumab, durvalumab, and avelumab) for the treatment of different types of cancer.

The combination of nivolumab and ipilimumab also appears to improve overall survival (OS) and progression-free survival (PFS) in patients with metastatic CRC and has an acceptable safety profile [118]. In patients with CRC, it has been demonstrated that a subgroup can benefit from immune checkpoint inhibitors. For example, Le et al. in a phase II clinical trial, after the administration of pembrolizumab, showed a partial objective response rate of 40% for DNA mismatch repair deficiency (dMMR) in CRC patients. Nivolumab was approved in 2017 for metastatic colorectal cancer (mCRC) refractory to fluoropyrimidine, oxaliplatin, and irinotecan with high microsatellite instability (MSI-H) and dMMR, in a dose of 3 mg/kg every two weeks. It achieved objective response rates (ORR) of (31%) and only 12% of patients presented secondary effects like fatigue, diarrhea, and pruritus. Currently, more questions than answers remain, including when to start treatment, the optimal sequence, and the optimal duration of treatment. What is certain is that the effectiveness of the treatment is only initial since as time passes immunological tolerance occurs that entails a mechanism of evasion of the immune system and therefore, a null therapeutic effect. It is also necessary to have more molecular tools or biomarkers to have a more precise evaluation of the expected response and the proper management of adverse effects and more multiple clinical trials that are in progress. An alternative in the field of immunotherapy is the granulocyte-macrophage colony-stimulating factor (GM-CSF) because it can enhance T cell proliferation and cytokine secretion of IFN-γ and IL-2. In CRC, GM-CSF improves the antibody response to CEA and inhibits metastasis [118]. Also, pancreatic cancer cells secrete GM-CSF, which can inhibit T-cell antitumoral activity by releasing IL-6 and IL-8. Whole-cell and antigen-specific vaccines have been developed. The first whole-cell vaccine was GVAX, which has GM-CSF. GVAX has been evaluated in metastatic CRC patients with or without chemotherapy showing an overall survival of 2.3 and 4.3 months with less toxic effects than conventional treatment [126]. Gene therapy has been applied for immunotherapy as encapsulation and delivery of plasmids DNA (pDNAs). Encoding PD-L1 and CXCL12 in a lipid calcium phosphate vector induces local expression in metastatic liver, reducing lymphoid structures associated with liver metastasis, progression, and immune evasion in CRC. This is a transitory expression. However, it can recruit CD8+ T-cell and activation in the metastatic niche. In murine models, this strategy improves survival by more than 70% [127]. B7H3 is a marker expressed on immune cells (such as APCs or macrophages) and tumor cells, and it has inhibitory roles on T cells, contributing to tumor cell immune evasion by immune tolerance. Some studies report that the upregulation of B7-H3 is closely related to lymph node metastasis in patients with CRC. Blocking B7H3 with a monoclonal antibody reduces the number of cancer-initiating cells and is, therefore, a potential new biomarker and therapeutic target for CRC. Furthermore, based on in vitro and in vivo experiments, it was shown that B7H3 in CRC cells positively regulates the expression of VEGFA and angiogenesis by activating the NF-κB pathway. Combination therapy of the B7H3 inhibitor 3E8 with bevacizumab inhibits tumor growth in mouse xenograft models. As such, combination therapy with B7H3 blocking and anti-angiogenesis compounds is expected to be applied for the clinical treatment of tumors. On the other hand, this strategy has also been shown to present activity in pancreatic cancer animal models, inhibiting the infiltration of CAFs that play a common role in immunosuppression by preventing the infiltration of T cells into tumors. When CAFs are depleted, these mice are susceptible to the antitumoral effects of both anti-CTLA-4 and anti-PD-1 antibodies. In pancreatic cancer there is a strong anti-inflammatory response by T helper type 17 (Th17) cells, T helper type 2 (Th2) cells, and reduction of APCs. There is also upregulation of immunoinhibitory receptors, such as CTLA-4 and PD-1, which improve therapeutic performance [126].

### 5.3. Vaccines

Vaccines in cancer immunotherapy are based on cancer metabolites that can induce a strong immune response by activation of T cells, and usually employ a vector for delivery. Dendritic cells (DCs) are the most important potent antigen-presenting cells in vivo, which prominently express costimulatory molecules that are uniquely able to induce primary immune responses. Most clinical trials using DC-vaccines are based on DC loaded with lysates of isolated CSCs. Recently, it has been reported by Li et al. that DC vaccination using lung CSC antigens induced MHC expression, cytokine production, lymphocyte infiltration, and long-term protection against prostate cancer. CD90^+^HepG2 cells were fused with DCs as a CD90^+^HepG2/DC vaccine. CD90^+^ is one of multiple antigens for CSC which by a fusion process with DC can present antigens to T cells, activating T cells to be cytolysis-specific CTLs to eliminate CSC. Also, DCs with CD44 or EpCAM peptides enhanced T cell stimulation thus resulting in the induction of cell cytotoxicity against human breast cancer and HCC [128] A CSC lysate has been used as a DC vaccine (Panc-1 and ALDH^high^) and has the ability to induce proliferation of T cell lymphocytes and B cells (IgG) which are capable of reducing tumor growth, the development of lung metastases, and increasing survival. Other immunotherapy alternatives in pancreatic cancer consist of a live-attenuated *Listeria monocytogenes* vaccine vector, which expresses mesothelin, a tumor-associated antigen expressed in a wide number of pancreatic cancers. This molecule activates mesothelin-specific T cells to eliminate cancer cells; patients with pancreatic cancer who underwent treatment with the vaccine had markedly prolonged survival [129]. This strategy has great benefits, such as generating scalable standardized vaccines by promoting humoral immunity-immunological memory with low toxic effects (compared with recombinant proteins or antibodies). However, to have the expected activity, it requires the co-stimulation of molecules and molecular signals that induce the clonal expansion of effector cells, which can sometimes be an obstacle to obtaining the desired effect.

### 5.4. Cell Therapy

Cancer progression is generally associated with impaired antitumor immunity and recruitment of regulatory cell populations to the tumor microenvironment. Cellular immunotherapy refers to the administration of living cells to a patient; this type of immunotherapy can be active, where the cells can stimulate an antitumor response in the patient (dendritic cells), or it can be passive, known as adoptive cell transfer (ACT), when the cells directly attack tumor cells. The cells that are mainly used are T lymphocytes, NK, and NKT, which are autologous or allogenic with or without modification. We summarize the recent research on cellular immunotherapy for destroyed CSC in CRC and pancreatic cancer [101,118].

### 5.5. Active Therapy

Success partial in CRC has recently been achieved with this therapy, as it has increased tumoral expression of an HLA-class I-associated β2- microglobulin molecule, an indicator of CD8+ T cell activity. In this study, three of 15 patients with metastatic CRC had stabilization or partial remission [118]. Although it turns out to be a promising approach, there is a more efficient strategy in which DCs are loaded with peptides, proteins, or mRNAs, controlling with greater precision the immune response generated. Autologous dendritic cells showed an anti-tumor effect in metastatic liver tumors in a phase II clinical trial [130]. Bagheri V et al. loaded DC with total gastric CSC mRNA expressing markers CD44, CD54, and EpCAM. These DC were able to induce expression of the IFN-γ gene and generate a cytotoxic effect after a 12-day coculture with T lymphocytes. Recent studies proposed the transformation of somatic cells into stem cells (iPS) by DC with transcription factor overexpression of CSC (NANOG, OKT4a, SOX2, c-MYC, and KLF4). For example, DCs loaded with NANOG peptides can generate immunological memory after vaccination and help the immune system to manage the plasticity of the CSC [28]. This strategy is quite promising since it is a specific target that will mainly affect CSC. Therefore, it will probably immunologically protect and expect to avoid the development of metastasis from CRC and pancreatic cancer. Allogeneic CD34+ hematopoietic stem cell transplantation is commonly employed in the treatment of blood-related malignancies after a regimen of lymphodepleting drugs. However, CD34+ stem cells play an important role during liver development and regeneration. Thus, we hypothesized that some human liver carcinomas (HLCs) might be derived from transformed CD34+ stem cells. We determined that a population of CD34+ stem cells functioned as liver CSCs (LCSCs). They were proposed as a marker of disease progression.

### 5.6. Passive Therapy

Natural killer (NK) cells participate in the innate immune response by targeting virus-infected, transformed, and allogeneic cells by NK cell receptors that are activated or inactivated to eliminate altered cells [131]. These receptors include NKp30, NKp44, NKp46, DNAX-activating molecule-I (DNAM-I), which can recognize ligands such as MICA, MICB, and a group of ULBPs. The ability of NKT cells in targeting CSC in colon adenocarcinoma lesions has been demonstrated. This is mainly due to higher expression of NKp30 and NKp44, both ligands of CSC for NKT cells [132]. The liver is the main site of CRC metastasis and shows an increased level of immature NKT cells, which indicates that NKT cells could be used as a strategy for cancer treatment [131]. Entolimod is a pharmacologically optimized flagellin derivative that can activate vertebral Toll-like receptor 5, and consequently activate the NF-kβ pathway. Moreover, this activation can inhibit the growth of tumoral cells expressing TLR5 while protecting normal tissues from radiation and ischemia-reperfusion injuries. It has been found that NK cells are critical for the activity of entolimod against liver metastases [131]. In another liver metastasis murine model, the alteration of commensal gut bacteria by antibiotics (vancomycin, neomycin, and primaxin) increases hepatic CXCR6+ NK cells in metastatic tumors. These NK cells also generate more interferon-γ (IFN-γ) [133]. Besides NK cells, T cells are also used in immunotherapy. Tregs (regulatory T cells) also benefit CRC patients as T cells marked with FoxP3+ in CRC correlate with patient survival and no development of metastases. Adoptive transfer of lymph node derived CD4+ TH1 cells in stage IV CRC patients improve complete remission of the disease. Adoptive T-cell transfer is T lymphocytes that are genetically modified ex vivo to express chimeric antigen receptors and recognize specific membrane proteins expressed on tumor cells [129]. This engineering of T-cells expressing chimeric antigen receptors (CARs) is another immunotherapy strategy. CARs are antigen-specific heavy and light chain antibodies genetically attached to cytoplasmic signaling molecules, such as 4-1BB, OX40, Luk, and TCR ζ-chain, which activate T cell cytotoxic activity without peptide presentation. This type of therapy is important when MHC class I is reduced. CAR T-cell technology was described more than twenty years ago and was a success with complete remission in hematological malignancies (B cells target) [118]. Modification of T cells for specific recognition of CEA, which has been widely identified in colorectal tumors, helps patients with advanced-stage CRC [118]. However solid tumors, such as breast, colorectal, prostate, and kidney, have not been nearly as successful. This strategy of treatment with CAR-T cells has significant limitations, accessibility, safety, and cost. A phase I clinical trial of immunotherapy for liver metastasis showed that patients receiving CAR-T cells modified with an anti-CEA receptor had increased necrosis or fibrosis in metastasis biopsies and a serum CEA decrease of 37%. Those patients were refractory to conventional therapy and showed more abundant levels of CAR-T cells in liver metastases than in normal liver. However, another study of CAR-T cells (HITM-SIT) showed the safety and activity of CAR-T infused by hepatic artery cells followed by selective radiotherapy (SIRT). In Table 4 are included some new strategies used for immunotherapy in liver and lung metastasis.

### 5.7. miRNA and siRNA

Therapies based on oligonucleotides are directed at target tumor suppressor genes and oncogenes in cancer cells to improve apoptosis and reduce cancer cell invasion. Oligonucleotide-based therapies include mRNA, siRNA, miRNA, and non-coding RNA [139]. KITENIN (KAI1 C-terminal interacting tetraspanin) is a member of the tetraspanin protein family that interacts with the C-terminal cytoplasmatic domain of KAI1. In CRC, KITENIN increases migration and invasiveness by recruiting Dishevelled (Dvl) and protein kinase Cδ (PKCδ); in contrast, its knockdown inhibits tumor metastasis by distorting actin arrangement and decreasing AP-1 target genes, such as MMP-1, MMP-3, and CD44. Intravenous administration of KITENIN shRNA reduces tumor growth and liver metastasis development in murine models of CRC [140]. An enhancer of zest homolog 2 (EZH2), a member of the Polycomb group (PcG) protein family, contributes to CRC acting as an oncogene. The sub-expression of EZH2 impairs the ability of CRC cancer cells to invade other sites and improves its apoptosis [141]. EZH2 is a target of miR-506. However, its expression is downregulated in CRC tissues. Overexpression of this miRNA significantly inhibits cell proliferation and metastasis of CRC by modulating the Wnt/β-catenin signaling pathway [142]. miR-155 is overexpressed in several cancers such as CRC; its overexpression is associated with poor prognosis and improves cell migration. HuR (ELAVL1) is a nuclear RNA binding protein (RBP) that has been associated with an increase of cell division by improving stabilization of COX-2, cyclin A, MMP-9, EGFR, and c-fos. HuR also promotes pro-inflammatory and angiogenic factors, such as TNF-α and VEGF, and is related to the migration process of cancer cells. Targeting miR-155-5p reduced HuR expression and migration of CRC cells by a union to 3-UTR in HuR mRNA [130]. miR-3653 expression is downregulated in CRC cells and tissues, which in turn improves metastasis. However, its overexpression is associated with less CRC cell migration and invasion, showing tumor suppression activity by inhibiting epithelial–mesenchymal transition. This is mediated by its binding to Zeb2 3’UTR [142]. A clinical trial (NCT03480152) used mRNA vaccination with epitopes from immunogenic neoantigens, predicted neoantigens, and mutations in tumor suppressor or driver genes for CRC liver metastasis [130]. For now, miRNAs can be used as a tool for diagnosis, prognosis, and as a therapeutic target as is mentioned in Table 5. Treatment can inhibit CSC functions and enhance sensitivity to conventional treatments and these results significantly improve the course of the disease. However, there is a coincidence in some cases microRNAs activate signaling pathways changes during cancer stages and when only one target of treatment is used it cannot considerably affect progression by inducing a resistance mechanism. For now, they turn out to be ideal candidates since they are specific to a cell type representing an ideal treatment for future research in the field.

### 5.8. Gene Therapy

Gene therapy implies the administration of a gene sequence to counteract a defect in target cells or eliminate a phenotype. Polo-like kinase 1 (PLK1) is a protein overexpressed in multiple human tumors as CRC. In general, PLKs have functions in mitosis ensuring the fidelity of checkpoint controls [130,143]. However, PLK1 es a biomarker of prognosis in CRC and is considered an oncogene of cell cycle progression. Thus, a siRNA has been developed against PLK1 encapsulated in lipid nanoparticles (TKM-080301), which has been used in a clinical trial for CRC liver metastases [143]. The administration of certain gene sequences for targeting liver metastases has been improved in recent decades. Rexin-G is a dominant-negative form of cyclin G1 released by non-replicable retroviral vector injection into the hepatic artery. Clinical trials are employing this strategy in CRC liver metastases and gemcitabine-refractory pancreatic cancer [143]. Rexin-G displays a cryptic collagen-binding motif on its gp70 surface membrane for targeting abnormal signature (SIG) proteins in the TME and encodes a dominant-negative mutant construct (dnG1) of human cyclin G1 (CCNG1), which in turn enters proliferating cancer cells and fibroblasts, and blocks the cell division cycle generating apoptosis [144]. Other viral gene therapies for metastatic pancreatic cancers are oncolytic adenovirus expressing IL-12 in combination with chemotherapy, vaccines of virus expressing CEA, mucin-1, and a triad of costimulatory molecules (TRICOM) (PANVAC-VF). This strategy is combined with GM-CSF at the vaccination site [144]. Recent studies have evaluated the use of CRISPR/Cas9 to replace native TCR which increases antigen sensitivity and specificity. Also, it can be used to knock out HLA from allogeneic cells to reduce the cost, time, and resources required to generate CAR T cells for every patient. Although it is still under investigation, the CART-cell strategy for solid tumors, probably in the next years, will be a great option to destroy solid tumors and prevent metastasis progression. Gene therapy in clinical practice is a viable alternative for monogenic diseases and cancer when standard treatments do not have good results. The design of new experimental vectors, increased efficiency, specificity of delivery systems, and a better understanding of the induction of the inflammatory response may balance improved safety expanding techniques in clinical applications. However, the knowledge and experience gained from a careful evaluation of the toxicity of these technologies also allow significant advances in the application of these methods. Therefore, gene therapy, like any new technology, needs more enlightening preclinical studies. In the future, there is a promise to apply these techniques in combination with other strategies and a greater percentage of clinical trials to evaluate its therapeutic potential.

### 5.9. Nanomedicine

Nanomedicine presents several advantages over chemotherapy such as enhanced permeability and retention in tumors, easy surface modification for tumor targeting by conjugating molecules, controlled drug release, the facility of structural and morphological modification, and the integration of various drugs [145]. Nanoparticles have diameters of 10 to 1000 nm. These systems have specific properties that modify the fate of the nanoparticle and loaded drug. Its application in cancer therapy has been widely explored and optimized for delivery systems for cytotoxic molecules. Nanoparticles can be developed from multiple elements, such as lipids, polymers, and carbon. Frugs can be loaded by different principles such as physical entrapment, covalent linking, and surface adhesion, which is selected according to drug properties as an active conformation. Attaching targeting moieties includes antibodies, nucleic acids, peptides, recombinant proteins, and aptamers [146]. However, the main interest in nanomedicine is that nanoparticles can be capable of eliminating cancer cells while avoiding toxicity in normal tissues [145]. Nanoparticles of chitosan-tripolyphosphate (CS-TPP) charged with IL12 have been developed for liver metastasis. This technology was able to release IL-12 in a sustained and acid-responsive way, with low toxicity compared to intravenous free administration, and improve recruitment and tumor infiltration of NK cells. This reduces the number and volume of CRC liver metastasis foci with a lower drug concentration than free IL-12 (0.1 μg/mg) [145]. NK012 polymeric micelle of SN38 (PEG-PGA) is a nanoparticle used in the treatment of cell lung cancer and metastatic CRC that is in a phase II trial [146]. Folic acid has tumor tissue specificity and is non-immunogenic. Linked in nanoparticles, folic acid has a high binding affinity to receptors expressed on tumor cells. There have been developing systems of RNA nanoparticles to carry therapeutic modules and folic acid ligands for specific CRC cell targeting. RNA packaging of bacteriophage phi29 DNA is used [147]. Melittin peptide presents immunomodulatory effects. In one study, nanoparticles were conjugated with α-melittin modulate liver stellate cells to become APCs, affecting cytotoxicity and generating a pro-inflammatory microenvironment to eliminate tumoral cells by infiltration of T and NK cells. Moreover, intravenous administration of α-melittin nanoparticles blocks metastasis formation and prolongs survival in liver metastases models [148]. There is a commercial compound based on nanoparticles, Onivyde, which is liposomal irinotecan (PEGylated) for metastatic pancreatic cancer. This drug was approved by the FDA in 2015, and allows prolonged circulation of irinotecan with reduced toxicity [146]. Nanoparticles are a very effective drug, protein, or nucleic acid delivery system. They present the advantages of high drug concentrations and specific targeting of cancer cells. However, their use against CSC is still complicated since, although an expected cytotoxic effect has been demonstrated in vitro in an in vivo model, accessibility in the tumor and the microenvironment play a role in the effectiveness of this therapeutic strategy. Furthermore, the current markers are not specific, and their expression level can change depending on the microenvironment. The current aim in this field is to develop multifunctional nanoparticles that can load multiple drugs simultaneously and that do not present long-term toxicity. To achieve this, a greater understanding of CSC and the metastatic niche is required in order to use this information to provide more efficient therapeutic strategies.

## 6. Conclusions

Cancer is typically detected late, at a time when the disease is in advanced clinical stages and many times, although detected in early stages, the disease can develop metastases. So far, there has been a greater understanding of the growth factors, cytokines, changes in the microenvironment, cells, microRNAs, and signaling pathways that orchestrate the premetastatic niche. However, much remains to be understood about the key regulatory mechanisms for the establishment of the metastatic niche and which of these can be used as biomarkers or therapeutic targets. Some markers and signaling pathways that coincide in lung and liver metastasis can be used to direct treatment or biomarkers used for diagnosis. Moreover, the results were very different even using a similar treatment approach. CSCs have taken a central role since they are characterized as the main cells responsible for metastasis and resistance to conventional treatments. A great effort has been made in recent years with various approaches to improve the diagnosis and treatment of cancer patients. One of the areas that continue to be one of the main approaches is immunotherapy, where to date, there is more and more clinical trials evaluating therapeutic targets using properties of CSC. In this work, we highlight the main findings and strategies used to treat patients with the most frequent metastases, which are liver and lung. In addition, we show that several groups seek to adopt strategies such as gene therapy, nanoparticles with peptides, or microRNA against CSC markers as targets with promising results. However, in this sense, it is still necessary to have specific markers and greater knowledge of mechanisms that allow CSCs to change expression depending on the microenvironment and therefore it is proposed that the disease, for now, is attacked by different targets to prevent resistance mechanisms from developing in the short term. The long-term effect of these strategies is still unknown, and therefore, we continue to evaluate the scope of these strategies in in vitro and in vivo models. Another important problem is that few biomarkers allow evaluating the response to treatment. Survival may change in some patients since the patient is sometimes expected to improve with the therapeutic strategies used but patients do not have the economic resources to carry out treatment completely as expected. On the other hand, it is necessary to better define the characterization of patients who are candidates for treatment, since in some patients, the clinical significance is not so relevant, and if they present significant side effects, they represent a high cost for families. Hopefully, the combination of some of the therapies and different biomarkers, genetics, and metabolites can contribute in the future to offer an effective and accessible treatment to change the course of metastatic disease and at the same time be specific for every patient’s needs.

## Figures and Tables

**Table 1:** Biomarkers of lung and liver metastasis.

Biomarker	Characteristic	Reference
Serum amyloid A (SAA)	-Main acute phase proteins expressed in the liver. -Circulating levels correlate with cancer progression and poor survival. -SAA3 in PDAC is associated with fewer differentiated tumors, greater migration, and the number of CD133+ CSCs.	[101]
Thrombospondin-2	-Detection of liver metastases. -Monitor therapy response.	[101]
DPC4	-Prognostic marker in PDAC and CRC. -Promotes EMT. -DPC4 loss is associated with metastatic disease.	[101]
ASPH	-Prognostic factor of PDAC. -Upregulated in metastatic PDAC.	[101]
Oncostatin M (OSM)	-Elevated in the serum of PC patients. -Associated with highly aggressive metastatic cancers, increased risk of tumor recurrence, and poor prognosis.	[101]
Non-alcoholic fatty liver disease fibrosis score (NFS)	-Includes age, diabetes/hyperglycemia, BMI, platelet count, albumin, and AST/ALT ratio. -Predicts survival in liver metastases.	[101]
Carcinoembrionary antigen (CEA)	-Predicts survival in liver metastasis during treatment.	[101]

**Table 2:** Conventional treatment for metastatic CCR and PDAC.

Treatment	Advantages	Limitations	Reference
Surgical resection	-Gold standard for liver metastases. -5-year survival of 33% in CRC liver metastases. -5-year survival is 27% in PDAC liver metastases. -Surgical mortality rate is <5%. The 5-year survival of 43% (21–62%) in PC lung metastases. -Better outcome in PDAC (40%) lung metastases.	-Limited liver resection. -Relapse occurred in 75% of patients in the first 2 years after surgery. -Liver resection is not available for all patients in PC. -5-year survival of 19.8% in PDAC lung multiple metastases.	[21,100,101,106]
Thermal ablation	-5-year overall survival of 19.9–70% in CRC lung metastases and 25–55% in liver metastases. -Minimal invasiveness, safety, equivalent local control, and survival to lung resection.	-More suitable for small-volume tumors (diameter ≤ 3 cm). -Aiming at a tumor-free margin of > 10 mm. -Recurrence of 46% after thermal ablation.	[107,108,109]
Regional hepatic chemotherapy	-Overall survival of 6 to 12 months. -Alternative for patients unable to hepatic resection or those with poor prognostic features. -Allows chemotherapy administration for a longer time. -10–20% of unresectable patients become resectable with chemotherapy	-70% of patients have residual metastases. -Pump placement by surgery.	[100,110,111]
Radiation	-Reduction in CEA. -Tumor volumes decrease in liver metastases. -Option for unresectable disease and for medically inoperable patients. -Survival is 70%, 46%, and 46% at 6, 12, and 18 months using selective internal radiation therapy (SIRT). -Local controlled disease is >70% using stereotactic body radiation therapy (SBRT).	-Requires chemotherapy for better response. -Tumor diameter of less than 6 cm.	[112,113]

**Table 3:** Immune checkpoint targets.

Molecule	Characteristics	Reference
ICOS (CD28)/ICOSL	-ICOS: ICOSL pathway provides key positive second signals that promote T cell activation, differentiation and effector responses, and T cell-dependent B cell responses.	[115]
CD27/CD70	-Ligation of CD27 by CD70 induces strong ubiquitination of TRAF and the activation of both canonical and non-canonical nuclear factor-kB (NF-kB) pathways. -Reduced regulatory CD4 T-cell numbers -CD70 is expressed in most primary human breast carcinomas and that its expression selectively correlates with lung metastasis.	[116,117]
GITR/GITRL	GITR enhances T-cell proliferation and that the absence of GITR is protective in several inflammatory disease models, which is attributed to an impaired effector T-cell function of T cells	[116]
PD-1/PDL-1 (PD-L2)	Members of CD28 and B7 families Induce tolerance by regulation of T-cell	[118]
BTLA/HVEM	BTLA may specifically downregulate Th1-mediated inflammatory responses Exerts inhibitory effects on B and T lymphocytes	[115]
CTLA4/CD80 (CD86)	An inhibitory, co-stimulatory molecule which interferes with the process of T cell activation Natural ligands of CTLA-4 are CD80 (B7-1) and CD86 (B7-2), which are both expressed by antigen-presenting cells (APCs).	[119]
B7H3	Tumor cell immune evasion promotes angiogenesis by upregulating VEGFA	[120]
B7H4	molecular biomarker associated with tumor progression and prognosis	[121]
B7H5/HVEM	Sub-expression of B7H5 is correlated with metastases and poor prognosis	[122]
LAG3/MHC II	Negatively regulates T-cell function, contributing to tumor escape.	[123]
TIM3/GAL9	Associated with immunosuppression and worse clinical outcome in multiple cancers.	[124]
TIGIT/Nectin-2	Inhibition of NK cell activation.	[125]
Indoleamine 2,3-dioxygenase (IDO)	Tolerance and suppressing T cell responses to MHC mismatched allografts, tumors, and self-antigens.	[115]

**Table 4:** Strategies of immunotherapy liver and lung metastasis cancer.

Strategy	Drug	Advances	Clinical Trials	Reference
**Anti-PD-L1**	Nivolumab Pembrolizumab	-Nivolumab has an objective response rate of 31% in metastatic CRC. -Pembrolizumab has a response rate of 40%.	NCT03307603	[118,134]
			NCT03832621	
			NCT04030260	
			NCT04575922	
			NCT02834052	
			NCT03265080	
	Atezolizumab Durvalumab Avelumab	-Suppress metastatic colonization on CRC cells. -Reduction of CEA levels.	NCT03721653	[118,134,135]
			NCT03256344	
			NCT03555149	
			NCT03193190	
			NCT03435107	
			NCT02734160	
			NCT03563144	
**GM-CSF Vaccine**	GVAX	-Enhance T cell proliferation and secretion of IFN γ and IL-2. -Overall survival of 2.3 and 4.3 months. -Less toxic effects.	NCT01417000	[118,126]
			NCT00727441	
			NCT02004262	
**Anti-B7H3**	Monoclonal antibody (3E8) and bevacizumab MGC018 DS-7300a	-Reduce CSC number. -Inhibits tumor growth and angiogenesis. -Depletion of cancer-associated fibroblasts.	NCT03729596	[120,136]
			NCT04145622	
**Dendritic Cells (DC) Vaccines**	DC loaded with lysates of CSC Peptides: CD90, CD54, CD44, EpCAM, Panc-1, ALDH, mRNA	-Induction of MHC expression, cytokine production (IFN γ), lymphocyte infiltration, proliferation T, and B lymphocytes.	NCT02615574	[128,130,137]
			NCT02503150	
			NCT00176761	
			NCT00558051	
			NCT00868114	
			NCT01410968	
**Mesothelin Vaccine**	Live-attenuated *Listeria monocytogenes* vaccine	-Activates mesothelin T cells in PC.	NCT03122106	[129]
			NCT03956056	
**DC Expressing CSC Transcription Factors**	NANOG, OKT4a, SOX2, c-MYC, KLF4	-Cellular immunological memory.	NCT00103142	[138]
**Natural Killer Cells**	Recognition of receptors in CSC: NKp30, NKp44	-Targeting of CSC.	NCT03008499	[131]
**CAR-T Cells**	Chimeric antigen receptors for specific proteins Anti-mesothelin Anti-CEA	-Increase in cell death. -Serum CEA decrease.	NCT01897415	[129]
			NCT01583686	
			NCT02416466	

**Table 5:** Novel miRNAs from CSC as target therapy.

miRNA	Disease	Characteristics	
miR-93	Pancreatic adenocarcinoma and lung metastases	Regulates microtubule dynamics by controlling YES1, CRMP2, and MAPRE1 expression.	[39,71]
miR-146a	Pancreatic adenocarcinoma and lung metastases	Diminishes in metastasis.	[72,73]
miR-200 family members: miR-194, miR-200b, miR-200c, and miR-429	Pancreatic adenocarcinoma	EMT facilitating invasion, targeting PTEN, EP300, and TGF-b/SMAD pathways.	[72,73,74]
miR-141 and miR-429	Pancreatic adenocarcinoma	Inhibit tumor development.	[75,76,77,78]
miR-10b	Pancreatic adenocarcinoma	Increases invasiveness in metastasis by the effect of EGF and TGF-β signaling.	[82,83,84]

## Data Availability

Data sharing not applicable.
